# ZZ/ZW sex chromosome system in the endangered fish *Lignobrycon myersi* Miranda-Ribeiro, 1956 (Teleostei, Characiformes, Triportheidae)

**DOI:** 10.3897/CompCytogen.v10i2.8435

**Published:** 2016-05-18

**Authors:** Alexandre dos Santos Rodrigues, Aline Souza Medrado, Débora Diniz, Claudio Oliveira, Paulo Roberto Antunes de Mello Affonso

**Affiliations:** 1 Universidade Estadual do Sudoeste da Bahia (UESB), Dep. Ciências Biológicas, Av. José Moreira Sobrinho, s/n, 45206-190 Jequié, BA, Brazil; 2 Universidade Estadual de Santa Cruz (UESC), Campus Soane Nazaré de Andrade, Rodovia Jorge Amado, Km 16, 45662-900 Ilhéus, BA, Brazil; 3 Universidade Estadual Paulista, Instituto de Biociências, Dep. Morfologia, 18618-000 Botucatu, SP, Brazil

**Keywords:** Evolution, female heterogamety, rDNA, sex determination, Triportheus

## Abstract

*Lignobrycon myersi* is an endemic fish species from a few coastal rivers in northeastern Brazil. Based on molecular evidence, *Lignobrycon myersi* and genera *Triportheus* Cope, 1872, *Agoniates* Müller & Troschel, 1845, *Clupeacharax* Pearson, 1924 and *Engraulisoma* Castro, 1981 were placed in the family Triportheidae. In the present work, we report the first cytogenetic data for *Lignobrycon myersi* to test the hypothesis that *Lignobrycon* and *Triportheus* are closely related. Studied specimens presented 2n=52 with 28 metacentric (m), 18 submetacentric (sm) and six subtelocentric (st) chromosomes for males and 27 m, 19 sm and 6 st for females, characterizing a ZZ/ZW sex chromosome system. The Z chromosome corresponds to the largest chromosome in karyotype while the W is about 50% smaller than the Z and largely heterochromatic. Terminal nucleolus organizer regions, GC-rich sites and 18S rDNA signals were detected on pair 14. However, additional 18S rDNA sites were observed in the W chromosome. The 5S rDNA was mainly detected on long arms of pair 7. The apparent synapomorphic chromosomal traits of *Triportheus* and *Lignobrycon myersi* reinforce their close phylogenetic relationship, suggesting that the ZZ/ZW chromosome system in both genera has arisen before cladogenic events.

metacentric

submetacentric

subtelocentric

## Introduction

*Lignobrycon myersi* Miranda-Ribeiro, 1956 is a small characin fish (about 11 cm in length) characterized by a compressed body with keeled coracoids, adapted to swim near the surface. The type-locality of *Lignobrycon myersi* is located in the Almada river basin, a costal drainage in Bahia (Castro and Vari 1990). Nearly 10 years later, this species was also collected in the nearby Contas river basin in Bahia (Castro and Jucá-Chagas 2008). Because of its narrow geographic range, associated with intensive environmental degradation (deforestation, pollution and impoundment), *Lignobrycon myersi* is currently listed in the IUCN Red List of Threatened Species of Brazil (Castro and Jucá-Chagas 2008).

Based on external morphology and osteological evidence, *Lignobrycon myersi* has been regarded as the only living sister-group of the elongate hatchetfish *Triportheus* Cope, 1872, composing the subfamily Triportheinae within Characidae (Malabarba 1998). Nonetheless, phylogenetic studies using DNA sequences of two mitochondrial and three nuclear genes revealed that this monophyletic group should be expanded and elevated to a family status (Triportheidae), including the following genera of tetras or freshwater sardines: *Agoniates* Müller & Troschel, 1845, *Clupeacharax* Pearson, 1924, *Engraulisoma* Castro, 1981, *Triportheus* and *Lignobrycon* (Oliveira et al. 2011).

Interestingly, *Triportheus* is one of the few fish groups in which sex chromosomes have probably appeared prior to the adaptive radiation of this genus (Artoni and Bertollo 2002). Thus, all species of *Triportheus* studied so far share a 2n = 52 and a ZZ/ZW sex chromosome system in which the W is remarkably smaller than Z chromosomes and usually carries 18S rDNA cistrons (Artoni and Bertollo 2002, Diniz et al. 2008a, 2009, Marquioni et al. 2013). Only *Triportheus venezuelensis* Malabarba, 2004 is differentiated by presenting nucleolus organizer regions (NORs) on Z chromosomes (Nirchio et al. 2007) (Table 1). This trend combined to the close relationship between *Lignobrycon* and *Triportheus* revealed by morphological and molecular analyses is appealing to cytogenetic studies in *Lignobrycon myersi*.

**Table 1.** Cytogenetic data in Triportheidae (species marked with “*” show synteny of 18S and 5S rNA).

Species	2n	Sex system	18S rDNA	5S rDNA	Reference
*Lignobrycon myersi*	52	ZZ/ZW	1 pair/W	2-4 pairs	present study
*Triportheus albus*	52	ZZ/ZW	1 pair/W	1 pair	Diniz et al. (2009); Marquioni et al. (2013)
Triportheus angulatus*	52	ZZ/ZW	2 pairs/Z/W	1 pair	Marquioni et al. (2013)
Triportheus auritus*	52	ZZ/ZW	2 pairs/W	5 pairs	Marquioni et al. (2013)
*Triportheus culter*	52	ZZ/ZW	1 pair/W	-	Falcão (1988)
*Triportheus guentheri*	52	ZZ/ZW	1 pair/W	1 pair	Diniz et al. (2009); Bertollo and Cavallaro (1992)
Triportheus nematurus*	52	ZZ/ZW	1 pair/W	1 pair	Diniz et al. (2008a); Marquioni et al. (2013)
Triportheus signatus*	52	ZZ/ZW	1 pair/W	1 pair	Diniz et al. (2009); Marquioni et al. (2013)
Triportheus trifurcatus*	52	ZZ/ZW	2 pairs/W	1 pair	Marquioni et al. (2013)
*Triportheus venezuelensis*	52	ZZ/ZW	1 pair/Z	-	Nirchio et al. (2007)

Therefore, the present work reports the first cytogenetic characterization in *Lignobrycon myersi* in order to understand the evolution of sex chromosomes within Triportheidae, particularly in relation to *Triportheus* species.

## Material and methods

**Figure 1.**
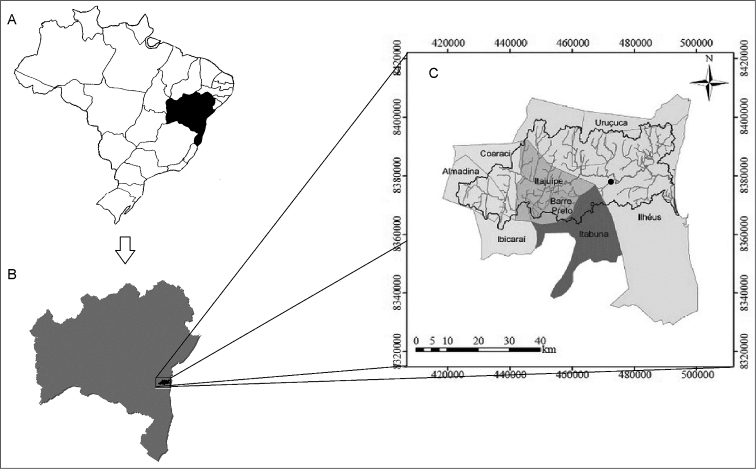
Map of Brazil (**a**), highlighting the state of Bahia (**b**) and collection site of *Lignobrycon myersi* in the Almada river basin (**c**).

Fourteen specimens of *Lignobrycon myersi* (4 males and 10 females) were collected in their type-locality in Braço (14°40'52"S/39°14'39"W) and Almada (14°39'35"S/39°13'24"W) Rivers, both belonging to the Almada River basin in the state of Bahia, northeastern Brazil (Fig. 1). Voucher specimens of *Lignobrycon myersi* were deposited under the code MBML 6400 in the fish collection of the Biology Museum Prof. Mello Leitão.

Metaphase chromosomes were obtained from anterior kidney cells as described by Netto et al. (2007), without mitotic stimulation. Chromosome spreads were stained with 5% Giemsa in phosphate buffer for karyotyping. Heterochromatin segments were visualized by C-banding (Sumner 1972) and active nucleolar organizer regions (Ag-NORs) were detected by silver nitrate staining (Howell and Black 1980). The GC- and AT-rich sites were identified by chromomycin A_3_ (CMA_3_) and 4,6-diamidino-2-phenylindole (DAPI), respectively (Schmid 1980).

The fluorescence *in situ* hybridization (FISH) was performed to map simultaneously 18S and 5S rDNA on chromosomes of *Lignobrycon myersi* according to Pinkel et al. (1986), with slight modifications and high stringency hybridization conditions (77%). The 18S rDNA probe was obtained from DNA of the red-eyed tetra *Moenkhausia sanctafilomenae* Steindachner, 1907 as described by Hatanaka and Galetti (2004), labeled with biotin-16-dUTP via nick translation using the BioNick Labeling System kit (Invitrogen) and signals were detected using avidin-fluorescein isothiocyanate (FITC) conjugate (Sigma). The 5S rDNA probe was obtained from DNA of the headstander *Leporinus elongatus* Valenciennes, 1850 (Martins and Galetti 1999), labeled with digoxigenin-11-dUTP via nick translation using Dig-Nick Translation Mix kit (Roche), and detected with anti-digoxigenin-rhodamine antibodies (Roche). Chromosomes were counterstained using DAPI (0.2 mg/mL) in Vectashield Mounting Medium (Vector) and slides were stored in a dark chamber up to analysis.

Metaphases were photographed using an Olympus BX-51 epifluorescence microscope equipped with digital camera and the software Image-Pro Plus® v. 6.2. The chromosomes were classified according to their morphology as proposed by Levan et al. (1964). The chromosomal pairs were arranged in karyotypes by decreasing size of chromosomes, as usually presented in cytogenetic reports of *Triportheus* (e.g. Diniz et al. 2008a).

## Results

**Figure 2.**
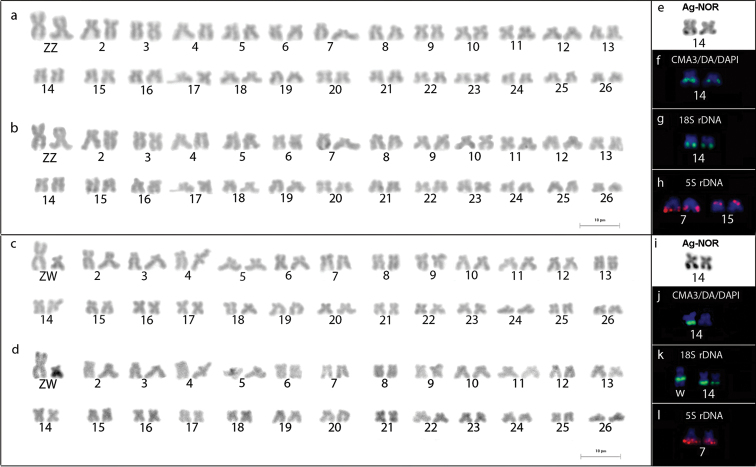
Karyotypes of male (**a, b**) and female (**c, d**) *Lignobrycon myersi* after Giemsa staining (**a, c**) and C-banding (**b, d**), bearing ZZ (1^st^ pair) and ZW sex chromosomes, respectively. On the right, the Ag-NOR bearing chromosomes (**e, i**), GC-rich region (CMA_3_^+^/DAPI^-^) (**f, j**), 18S rDNA (**g, k**) and 5S rDNA (**h, l**) in males (**e–h**) and females (**i–l**).

Both males and females of *Lignobrycon myersi* shared a modal diploid number of 2n = 52. The chromosomal pairs of males were homomorphic (Fig. 2a), being composed of 28 metacentric (pairs 1, 2, 10, 12, 15, 18–26), 18 submetacentric (pairs 3, 4, 6–9, 11, 14, 16) and six subtelocentric (pairs 5, 13, 17) chromosomes. In turn, females were differentiated by the presence of a single metacentric chromosome equivalent to pair 1, besides a small submetacentric chromosome, absent in males (Fig. 2c). Therefore, *Lignobrycon myersi* is characterized by the occurrence of differentiated sex chromosomes of ZZ/ZW type, being the Z chromosomes equivalent to the first and largest chromosomal pair.

The heterochromatin segments were distributed in small amounts over pericentromeric and terminal regions of some chromosomal pairs (Fig. 2b). The small submetacentric W chromosome was mostly heterochromatic with euchromatin restricted to terminal region of short arms (Fig. 2d).

The silver staining revealed a single NOR-bearing submetacentric pair (14^th^) with heteromorphic marks at terminal regions on long arms in both sexes (Fig. 2e, i). Similarly, GC-rich sites (CMA_3_^+^ and DAPI^-^) were coincident with Ag-NORs (Fig. 2f, j) and also characterized by size heteromorphism since fluorescent signals were occasionally absent in one of the homologues (Fig. 2j).

The FISH with 18S rDNA probe confirmed the presence of NORs on pair 14 as well as the size differences between clusters in homologous chromosomes (Fig. 2g, k), as verified by silver nitrate and CMA_3_ staining before. In addition, 18S rDNA sequences were also detected at interstitial region of the W chromosome (Fig. 2k).

The 5S rDNA cistrons were located at a terminal position on the long arms of a subtelocentric chromosomal pair (7^th^) in both sexes (Fig. 2h, l). Male specimens were further characterized by an additional 5S rDNA signal on short arms of subtelocentric chromosomes from pair 15 (Fig. 2h). However, it is not possible to state if these additional sequences are male-specific because of the reduced sampling (four specimens) in FISH experiments.

## Discussion

In spite of advances in cytogenetic studies of tropical ichthyofauna over the last decades, chromosomal reports about native fish populations from hydrographic basins in northeastern South America are recent and scarce (Bitencourt et al. 2012, Almeida et al. 2013, Nascimento et al. 2014, Medrado et al. 2015).

The karyotypic macrostructure of *Lignobrycon myersi* is similar to that reported in *Triportheus* in as much as both genera share 2n = 52 biarmed chromosomes and a differentiated ZZ/ZW sex chromosome system (Table 1). Even though ZZ/ZW sex chromosomes are relatively frequent in neotropical fishes, they have evolved independently in most lineages (Cioffi et al. 2012). *Triportheus* was regarded as the only exception in which the presence of heteromorphic sex chromosomes could be considered an apomorphic trait based on some peculiar features (Artoni et al. 2001, Artoni and Bertollo 2002, Diniz et al. 2009 among others), which are now also identified in *Lignobrycon myersi* for the first time.

Namely, the Z chromosome of *Lignobrycon myersi* corresponds to the largest metacentric chromosome of the karyotype, a feature also observed in *Triportheus* (Artoni et al. 2001, Artoni and Bertollo 2002, Nirchio et al. 2007, Diniz et al. 2008a). Even though the W chromosome in *Triportheus* species is invariably smaller than the Z chromosome, a comparative analysis of the relative length of the W chromosome in relation to the Z chromosome (WRL) revealed three trends in this genus, as follows: (1) species with WRL higher than 60%, (2) species with WRL ranging from 40 to 60%, and (3) species with WRL below 40% (Diniz et al. 2008a). Using the same parameters, the W chromosome of *Lignobrycon myersi* is about 50% smaller than Z, being classified as a medium-sized W chromosome as reported in *Triportheus nematurus* Kner, 1858, Triportheus prope. signatus, and *Triportheus guentheri* Garman, 1890 (Falcão 1988, Sánchez and Jorge 1999, Artoni et al. 2001, Diniz et al. 2008a).

It should be pointed out that *Triportheus guentheri* occurs in the São Francisco river basin (Reis et al. 2003). This basin shares a common evolutionary history with coastal rivers in Bahia, being isolated from each other by Espinhaço Range (Chaves et al. 2015). Therefore, the presence of a medium-sized W chromosome (see Diniz et al. 2008a, 2008b) might be a basal feature in Triportheidae. The similarity in sex chromosome structure and adjacent geographic range suggest a close phylogenetic relationship between *Lignobrycon myersi* and *Triportheus guentheri*, which remains to be investigated.

Another trait that reinforces the conserved structure of sex chromosomes in *Triportheus* is the presence of 18S rDNA on the W chromosomes of all species (Artoni and Bertollo 2002) but *Triportheus venezuelensis* (Table 1). Moreover, the 18S rDNA on the Z chromosome of *Triportheus venezuelensis* was not stained by silver nitrate suggesting that it is an inactive rDNA cistron (Nirchio et al. 2007).

In turn, *Lignobrycon myersi* was characterized by a single pair of Ag-NORs located at terminal regions of pair 14. Single NORs are widespread in several fish taxa (Gornung et al. 2013), but rarely found in Characidae (e.g. Medrado et al. 2015), thereby providing additional support to the removal of *Lignobrycon myersi* and *Triportheus* from this family (Oliveira et al. 2011). The location of NORs in autosomes allowed differentiating *Lignobrycon myersi* and *Triportheus* species, since they are differentially located on long and short arms, respectively. However, this distinctive position of 18S rDNA cistrons might either be a result of actual chromosomal rearrangements (transpositions or inversions) or a technical artifact related to differences in condensation of chromosomes or biased measurements by each author.

On the other hand, the FISH with 18S rDNA probes showed that, similarly to other *Triportheus* species, *Lignobrycon myersi* also bears NORs on the W chromosome, even though they were inactive in studied samples (i.e. undetected by silver nitrate staining) (Fig. 2k). This result strengthens that the origin of differentiated sex chromosomes has taken place before the diversification in Triportheidae, instead of being restricted to the origin of *Triportheus* (Diniz et al. 2009). Putatively, during the evolutionary history of *Lignobrycon myersi*, the 18S rDNA sequences may have partially degenerated and thus inactivated (see Wilson and Makova 2009) while remaining functional in *Triportheus*, thus detectable by silver nitrate staining. To confirm this suggestion, a larger number of individuals should be cytogenetically analyzed for Ag-NORs at different periods, since this apparent inactivation can either be a transitory cell state or a polymorphic condition.

Large amounts of heterochromatin are a common feature of W and Y chromosomes in animals (Wilson and Makova 2009, Livernois et al. 2012), being clearly observed in *Lignobrycon myersi* and several species of *Triportheus* (e.g., Artoni and Bertollo 2002, Diniz et al. 2008a, 2008b, Cioffi et al. 2012). Thus, the heterochromatinization of W chromosomes seems to be associated with degeneration followed by chromosomal reduction during evolution of sex chromosomes (Bertollo and Cavallaro 1992, Diniz et al. 2008b). Indeed, Z and W chromosomes of species in early stages of sex chromosome differentiation, such as ratite birds (ostrich, emu and allies), are similar in both size and content of heterochromatin/euchromatin (Livernois et al. 2012) even though the relationship between age and sex chromosome degeneration is currently under debate (Bachtrog et al. 2014).

In spite of sharing a similar C-banding pattern, the base composition of repetitive DNA within heterochromatin segments of W chromosomes in *Triportheus* and *Lignobrycon myersi* seems more variable. While the GC-rich heterochromatic regions (CMA_3_^+^) in *Lignobrycon myersi* were interspersed to Ag-NORs only, as reported in some species of *Triportheus* (Artoni & Bertollo, 2002), conspicuous CMA_3_^+^ signals were reported in both autosomal NORs and W chromosomes of other species like *Triportheus nematurus* (Diniz et al. 2008a). In fact, the GC-rich blocks in *Lignobrycon myersi* were so reduced that no fluorescent signal was detected in homologues of some metaphase spreads (Fig. 2j).

The most divergent chromosomal trait observed in *Lignobrycon myersi* and other triportheids refers to the distribution of 5S rDNA sites, thereby demonstrating the evolutionary dynamics of this class of ribosomal genes and their potential to cytotaxonomy (Affonso and Galetti 2005, Molina et al. 2012). Most *Triportheus* species analyzed so far share syntenic 18S and 5S rDNA cistrons (Table 1), regarded as an ancestral trait for this genus (Diniz et al. 2008a; Marquioni et al. 2013). The non-synteny of both rDNA classes in *Lignobrycon myersi* (Fig. 2g-h, k-l) supports this inference, suggesting that transposition of 18S rDNA cistrons to adjacent position of 5S rDNA cistrons or vice-versa has taken place after the differentiation of *Lignobrycon* and *Triportheus*. Moreover, *Triportheus* species usually present 5S rDNA on short arms of a single sm pair (Marquioni et al. 2013), while *Lignobrycon myersi* was characterized by conspicuous signals on long arms of pair 7 in both sexes and on short arms of a second pair in male samples.

Therefore, the location of 5S rDNA sites in *Lignobrycon myersi* should represent an autopomorphic trait, even though the numerical polymorphism in 5S rDNA signals should be further investigated. On the other hand, the lack of synteny between 18S and 5S rRNA genes has been also reported in *Triportheus guentheri* from São Francisco river basin, reinforcing the putative evolutionary relationship between this species and *Lignobrycon myersi*, as abovementioned.

In conclusion, the cytogenetic results agree with morphological (Malabarba 1998) and molecular evidence (Oliveira et al. 2011) by revealing a series of synapomorphies between *Lignobrycon myersi* and *Triportheus* that reinforce their close evolutionary relationship. Moreover, present results suggest that ZZ/ZW sex chromosomes have evolved in the basal Triportheidae lineage, including other taxa than *Triportheus*. In this sense, further cytogenetic studies in other genera allocated in Triportheidae (*Agoniates*, *Clupeacharax* and *Engraulisoma*) by Oliveira et al. (2011) are strongly encouraged. Similarly, chromosomal analyses in other populations of *Lignobrycon myersi* (e.g. Contas River) can be useful to evaluate interpopulation differences or the existence of cryptic forms that should be prioritized for conservation.
